# The Relay/Converter Interface Influences Hydrolysis of ATP by Skeletal Muscle Myosin II*

**DOI:** 10.1074/jbc.M115.688002

**Published:** 2015-11-19

**Authors:** Marieke J. Bloemink, Girish C. Melkani, Sanford I. Bernstein, Michael A. Geeves

**Affiliations:** From the ‡ School of Biosciences, University of Kent, CT2 7NJ Canterbury, United Kingdom and; the § Department of Biology, Molecular Biology Institute, and SDSU Heart Institute at San Diego State University, San Diego, California 92182-4614

**Keywords:** actin, fluorescence, homology modeling, kinetics, muscle, myosin, protein structure-function, sequence alignment

## Abstract

The interface between relay and converter domain of muscle myosin is critical for optimal myosin performance. Using *Drosophila melanogaster* indirect flight muscle S1, we performed a kinetic analysis of the effect of mutations in the converter and relay domain. Introduction of a mutation (R759E) in the converter domain inhibits the steady-state ATPase of myosin S1, whereas an additional mutation in the relay domain (N509K) is able to restore the ATPase toward wild-type values. The R759E S1 construct showed little effect on most steps of the actomyosin ATPase cycle. The exception was a 25–30% reduction in the rate constant of the hydrolysis step, the step coupled to the cross-bridge recovery stroke that involves a change in conformation at the relay/converter domain interface. Significantly, the double mutant restored the hydrolysis step to values similar to the wild-type myosin. Modeling the relay/converter interface suggests a possible interaction between converter residue 759 and relay residue 509 in the actin-detached conformation, which is lost in R759E but is restored in N509K/R759E. This detailed kinetic analysis of *Drosophila* myosin carrying the R759E mutation shows that the interface between the relay loop and converter domain is important for fine-tuning myosin kinetics, in particular ATP binding and hydrolysis.

## Introduction

The myosin family consists of at least 35 different classes (1), which display a wide range of activities such as muscle contraction, phagocytosis, cell motility, tension maintenance, and vesicle transport (2). Class II myosins are responsible for muscle contraction in higher eukaryotes and produce a variety of modes of muscle contraction. The type of myosin heavy chain isoform predominantly expressed results in differing ATPase activity, unloaded shortening velocity, and contractile force (3). Although all myosins appear to undergo the same ATP-driven cycle of interaction with actin, known as the cross-bridge cycle, it is still not well understood how they are each finely tuned to their different tasks (4).

Key events in the cross-bridge cycle are the actin·myosin power stroke, which is thought to be closely coupled to the phosphate release step, and its reversal when detached from actin (the recovery stroke), which is coupled to the closure of switch 2 and the ATP cleavage step (5). Central to both power stroke and recovery stroke is the swing of the light chain binding domain or lever arm that amplifies small movements in the nucleotide-binding site to produce a movement of the end of the lever arm by up to 10 nm. The nucleotide site and the lever arm are linked through two structural elements: the converter domain and the relay loop-helix. Understanding the transmission of information through this pathway (nucleotide pocket – relay helix – converter domain – lever arm) is a goal of this work.

Details of the interaction between the nucleotide site and the lever arm movement are best described for the recovery stroke, where crystal structures of myosin, complexed with analogues of ATP and ADP·P_i_, have revealed high resolution structures of the pre- and post-recovery state conformations (6, 7) and allowed detailed molecular dynamic simulations of the transition between the two (8). In essence, the presence of the γ-P_i_ in the nucleotide binding pocket allows the switch-2 element (SW2)^4^ to close and form a stable interaction with the P_i_. SW2 is at one end of the relay helix, and the movement of SW2 onto the P_i_ causes a twisting and bending of the relay helix such that the distal end of the relay helix goes through a large movement. The distal end of the relay element (relay loop) is in close contact with the converter (9), and as the relay moves, the converter moves with it and the lever arm amplifies the movement to a 5–10-nm translation of the myosin tail. The power stroke is to a first approximation considered a reversal of this recovery stroke, whereas myosin is attached to actin and is coupled with loss of P_i_ from the nucleotide pocket. The lack of any high resolution structures during the power stroke means the molecular details of this event are less well defined. The contact between the relay helix and the converter is thus a central element in the efficient transmission of information between the nucleotide pocket and the lever arm, which we probe here.

*Drosophila melanogaster* is a classical model system for studying many eukaryotic proteins because of its well developed tractable genetics. Muscle myosin II has been well studied in *Drosophila* because its function can be analyzed at many different levels of organization, allowing an integrative approach from the isolated myosin molecule through to the operating muscle in the adult fly (10, 11). Furthermore, all muscle myosin isoforms in *Drosophila* are expressed from a single gene (*Mhc*) using alternative splicing of a set of six exons, four of which are in the motor domain (12, 13). This allows expression of at least 21 different myosin isoforms and provides an exciting system to explore the role of the different exons in tuning the function of individual myosins for their biological role.

**FIGURE 1.**
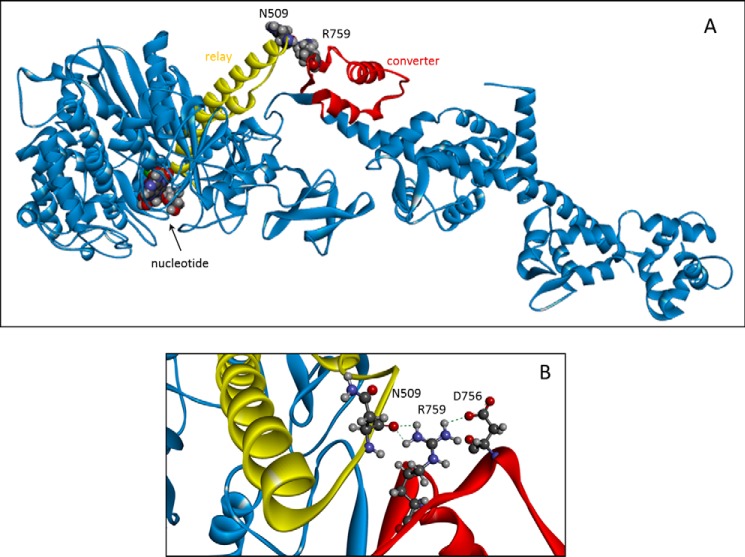
**Overview of myosin S1 with relay area (*yellow*) and converter domain (*red*) indicated.** *A,* residues 509 (relay loop) and 759 (converter) are shown as space-filling models. The nucleotide is also shown at the opposite end of the relay helix. *B,* close-up of the interface between the relay loop and converter domain with converter residue Arg-759 interacting with variable relay loop residue Asn-509 and converter residue Asp-756. Homology model of the *Drosophila* IFI myosin was built using the coordinates of PDB 1KK8 (actin-detached post power-stroke state) as a template.

This work will focus on two alternatively spliced exons, exon 11 and exon 9. Exon 11 encodes for the converter region residues 724–764 of chicken fast skeletal myosin II or residues 721–761 of *Drosophila*, whereas exon 9 represents the “relay helix-loop-helix domain” or “relay domain” and corresponds to residues 472–528 of chicken myosin II (or residues 469–525 of *Drosophila*). The two variable regions encoded by exon 11 and 9 interact with each other, as shown in Fig. 1*A*. Exchanging the converter region (exon 11) between the fast indirect flight muscle (IFM) and the slow embryonic (EMB) isoforms alters the mechanics and kinetics of the IFM and EMB fibers toward those of the donor isoform (14). Exchange of the relay domain (exon 9) between IFM and EMB does not alter the functional properties of the fast IFM myosin but does change the slow EMB myosin properties, *i.e.* reduced ATPase activity, increased actin affinity, eliminated actin motility, induced defects in myofibril assembly, and rapid degeneration of muscle structure (15). Using motor domain or S1 fragments of chimeric constructs (IFI-9b and EMB-9a), transient kinetics studies also demonstrated reduced values for the rate constant of ATP-induced actomyosin dissociation and ADP affinity for actin·S1 compared with both wild-type IFM and EMB S1 (16). Homology models indicated an important role for converter residue Arg-759 in maintaining close contact (<5 Å) between the converter and the relay region (17), as shown in Fig. 1*B*.

As predicted from the modeling studies, the R759E mutation in the myosin of the indirect flight muscle of *Drosophila* resulted in major defects, including loss of flight, loss of myofibril stability, and reduction of maximal power output (58%) (18). Studies with the isolated mutant myosin revealed reduced myosin ATPase activity and reduced actin velocity in *in vitro* motility assays (19). Following on from the work on the single mutation, Kronert *et al.* (17) reported that the double mutation R759E/N509K rescued many of the original R759E defects, including flight ability, muscle ultrastructure, myosin ATPase and *in vitro* motility. These results confirm the important role of Arg-759 in maintaining the interaction between the converter and relay loop in IFI myosin and its contribution to the high muscle power and optimal flight performance in *Drosophila.* The exact role of Asn-509 in this process is currently not clear.

Here we follow up on the recent work on the myosin R759E and R759E/N509K mutations. We have isolated the S1 motor domain from these mutant myosins and completed a biochemical kinetic study of the key events in the cross-bridge cycle. We find the effects of single and double mutations on the S1 ATPase activities are similar to full-length myosins, but surprisingly, we see little effect of the R759E mutation on the kinetics of the individual steps in the actin·S1 ATPase cycle. The exception is an inhibition of the ATP hydrolysis/recovery stroke by R759E, which is recovered in the R759E/N509K double mutation. The biological implications of this observation are discussed.

## Experimental Procedures

### 

#### 

##### Proteins

Monomeric G-actin was prepared from chicken skeletal muscle after several steps of polymerization-depolymerization as recently described (17). Filamentous actin was prepared from G-actin and used for actin-stimulated ATPase activity (17, 20). Muscle myosin was isolated through multiple purification steps from microscopically dissected dorso-longitudinal indirect flight muscles of ∼200 transgenic flies. Myosin subfragment 1 (S1) was prepared by chymotrypsin digestion as reported previously, and the concentration of purified S1 was determined by spectrophotometry (16, 20).

##### Steady-state ATPase Activity

Fresh S1 prepared after chymotrypsin digestion of myosin was used for steady-state ATPase activity. S1 ATPase activities were determined using [γ-^32^P]ATP as described (17, 20). Basal Ca^2+^-ATPase was determined as per full-length myosin and previously reported for S1 (15, 16). Both basal Mg^2+^ and actin-activated Mg^2+^-ATPase were determined using Mg^2+^-ATPase buffer without KCl (10 mm imidazole, 0.1 mm CaCl_2_, 1 mm MgCl_2_, 1 mm [γ-^32^P]ATP) as reported previously (20). Basal Mg^2+^-ATPase activities obtained in the absence of actin were subtracted from all actin-activated data points. Actin-activated *V*_max_ and *K_m_* values for actin were obtained by fitting all data points from several preparations of wild-type myosin S1 or mutant S1 with the Michaelis-Menten equation using SigmaPlot. Values were averaged to give mean ± S.D. Statistical differences of Ca^2+^-ATPase, Mg^2+^-ATPase, *V*_max_, *K_m_*, and catalytic efficiency between wild-type, mutant, and suppressor S1 were carried out using Student's *t* tests as described previously (20).

##### Flash Photolysis System

Flash photolysis was used to measure the transient kinetics of the *Drosophila* myosin S1 because of the small amounts of protein available (21, 22). By measuring changes in light scattering, the ATP-induced dissociation of the acto-S1 complex was followed after ATP release from caged ATP using a laser pulse, whereas changes in fluorescence were used to measure the dissociation of nucleotide from S1 alone. For the fluorescence measurements, S1 was incubated with a coumarin-labeled ADP analogue (deac-eda ADP) before displacement by ATP (23, 24). A low salt buffer was used for the light scattering experiments (30 mm KCl, 5 mm MgCl_2_, 20 mm MOPS, and 4 mm DTT, pH 7.0) with 1 μm actin, 1–3 μm S1, 500 μm caged ATP, 10 mm DTT, and either apyrase (2 units/ml), for ATP-induced dissociation of acto-S1 or ADP (various concentrations), and a glucose-hexokinase system (0.03 units/ml hexokinase, 1 mm glucose, and 100 μm Ap_5_A) for determination of *K*_AD_ as described previously (25). The light scattering traces were fitted with single exponentials to determine the *k*_obs_. Hyperbolic plots of the *k*_rel_ (*k*_obs_/*k*_0_) *versus* ADP concentration were fitted with an equation derived from Scheme 2 (*k*_obs_ = *K*_1_*k*_+2_([ATP]/(1 + [ADP]/*K*_AD_)) to determine *K*_AD_ (see under “Analysis of Transient Kinetics”).

##### Stopped-flow Measurements

Measurements were performed with a High-Tech Scientific SF-61 DX2 stopped-flow system at 20 °C. Intrinsic tryptophan fluorescence was measured using a 295 nm excitation wavelength and observed through a WG320 filter (26). All stated concentrations of reactants are those after mixing in the stopped-flow observation cell, unless otherwise specified. Stopped-flow data were analyzed using the Kinetic Studio software provided by TgK Scientific, as well as with Origin (Microcal).

##### Analysis of the Transient Kinetics Data

**SCHEME 1.**
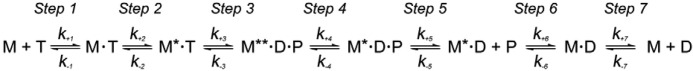
**Interaction of S1 with ATP and ADP.** S1, ATP, and ADP are represented as *M, T,* and *D,* respectively. * indicates the different levels of tryptophan fluorescence and represents different conformational states of the myosin.

**SCHEME 2.**
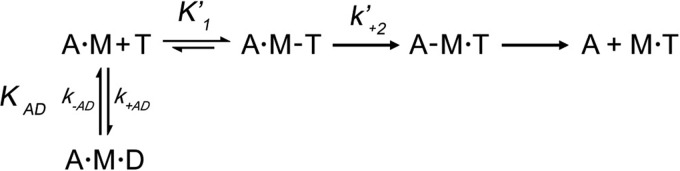
**Interaction of S1 with actin, ATP, and ADP.** Myosin, actin, ATP, and ADP are represented as *M, A, T,* and *D,* respectively. *Dashed* interactions represent a weakly bound complex, and *dotted* interactions represent strongly bound states. Cross-bridge detachment from the rigor state (*A*·*M*) involves the complex binding ATP, governed by the association constant, *K′*_1_, followed by the rate-limiting isomerization, *k′*_+2_, after which actin-myosin affinity becomes weak, and the complex separates rapidly.

Without actin present, the kinetics data of S1 with ATP (T) or ADP (D) were analyzed using the seven-step model described previously (27). The rate constants *k*_+_*_i_* and *k*_−_*_i_* are the forward and reverse rate constants. and *K_i_* (= *k*_+_*_i_*/*k*_−_*_i_*) represents the equilibrium constant of the *i*th step of the reaction (Scheme 1). In the presence of actin, the stopped-flow kinetics data were analyzed based on Scheme 2. To determine the ADP affinity in the presence of actin (*K*_AD_) Equation 1 was used,

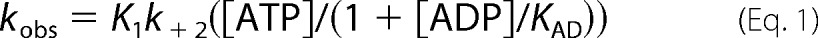
 where *k*_obs_ is the observed rate constant for the ATP-induced dissociation of acto-S1; *K*_1_*k*_+2_ is the second-order rate constant for ATP binding to acto-S1; and *K*_AD_ is the equilibrium dissociation constant for the binding of ADP to acto-S1. To determine the relative rate constant (*k*_rel_), the equation *k*_rel_ = *k*_obs_*/k_o_* was used (see Fig. 4), where *k_o_* is the measured rate constant *k*_obs_ when [ADP] = 0.

##### Homology Modeling

Three-dimensional homology models were generated for the *Drosophila* wild-type IFI myosin and converter mutant motor domains using the SWISS-MODEL (28) automatic comparative protein modeling server as described previously (16, 28). Briefly, the primary sequences of the *Drosophila* wild-type IFI and converter mutants were aligned pairwise with the sequence of four scallop myosin crystal structures as templates (PDB codes 1KK8, 1QVI, 1S5G, and 1SR6 using the ClustalW alignment protocol, and the alignments were submitted to the alignment interface of SWISS-MODEL (30, 31)). The scallop myosin structures used as templates represent various conformational states of myosin during the cross-bridge cycle; the actin-detached state contains ADP-BeF*_x_* (PDB code 1KK8); the pre-power stroke state contains ADP-VO_4_ (PDB code 1QVI), a conformation that contains partially bound ADP-SO_4_ (PDB code 1S5G) and the near-rigor state of myosin (PDB code 1SR6), which does not have a nucleotide in the binding pocket. Although scallop templates represent multiple myosin states, they have an alanine at the equivalent position of Asn-509 of *Drosophila* and may therefore not be the ideal templates to predict the side chain conformation of Asn-509. Chicken smooth muscle myosin has an aspartate at the equivalent position of Asn-509. Therefore, we also used chicken smooth structures as templates to build homology models for wild-type IFI, R759E, and N509K/R759E (PDB codes 1BR1, 1BR2, 1BR4, and 3J04) as smooth chicken myosin and *Drosophila* IFI share >50% of the myosin head domain sequence. The nucleotide binding pocket of smooth muscle myosin contains MgADP-AlF_4_ (PDB codes 1BR1 and 1BR2) and MgADP-BeF*_x_* (PDB code 1BR4) representing the pre-power stroke state (32). The 3J04 template is derived from phosphorylated smooth chicken myosin in the presence of ATP (33). Overlay of crystal structures of myosin head domains of scallop (PDB code 1SR6) and *Drosophila* embryonic myosin (PDB code 4QBD) was done using the Visual Molecular Dynamics (VMD) software (34).

## Results

### 

#### 

##### Steady-state ATPase Activity Is Reduced for R759E but (Partially) Restored for R759E/N509K

**FIGURE 2.**
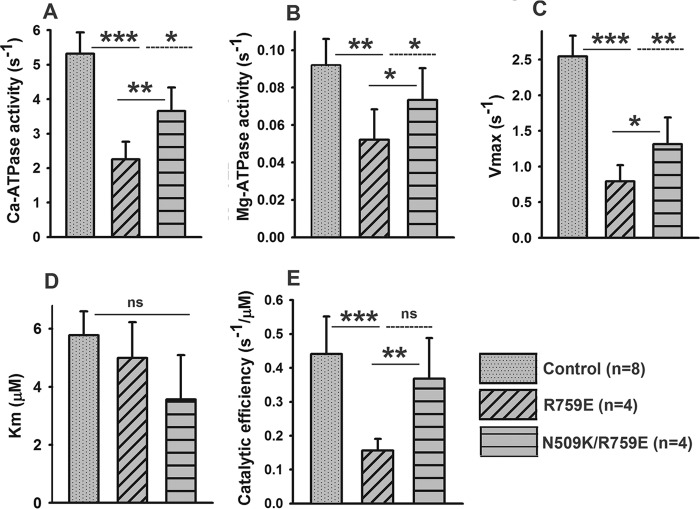
**Steady-state ATPase activity of wild-type control, single mutant R759E, and double mutant R759E/N509K *Drosophila* myosin.** Basal Ca^2+^-ATPase activity (*A*), basal Mg^2+^-ATPase activity (*B*), actin-stimulated Mg^2+^-ATPase activity *V*_max_ (*C*), actin affinity relative to Mg^2+^-ATPase (*K_m_*) (*D*), and catalytic efficiency (*E*) were determined as described under “Experimental Procedures.” Notations *above* histograms indicate the level of statistically significant differences (*, *p* < 0.05; **, *p* < 0.01; ***, *p* < 0.001; *ns,* not statistically significant). Significant differences were assumed for *p* < 0.05.

Basal Ca^2+^- and Mg^2+^-ATPase activity of wild-type myosin S1 and the single and double mutants S1(R759E) and S1(R759E/N509K) were measured according to established procedures (20). As shown in Fig. 2, *A* and *B,* the converter mutant R759E displayed a 2-fold reduction in basal Ca^2+^-ATPase (2.26 ± 0.51 s^−1^) and Mg^2+^-ATPase (0.052 ± 0.016 s^−1^) compared with wild-type IFI S1 (5.32 ± 0.62 and 0.092 ± 0.014 s^−1^ respectively). The double mutant (a potential suppressor) R759E/N509K was able to significantly restore both basal Ca^2+^-ATPase (3.65 ± 0.69 s^−1^) and Mg^2+^-ATPase (0.073 ± 0.017 s^−1^). As shown in Fig. 2*C,* the maximal actin-activated activity of R759E dropped to 30% of wild-type levels (*V*_max_ = 0.79 ± 0.23 and 2.54 ± 0.29 s^−1^, respectively), whereas *V*_max_ of R759E/N509K showed 52% of wild-type activity (*V*_max_ = 1.31 ± 0.38 s^−1^). Furthermore, the *V*_max_ of the suppressor was significantly higher (1.31 ± 0.38 *versus* 0.79 ± 0.23 s^−1^) than the *V*_max_ of the converter mutant R759E (Fig. 2*C*). Unlike full-length myosin *K_m_* (17), no significant change was found in the *K_m_* values for actin between wild-type, R759E, or R759E/N509K S1 (5.77 ± 0.83, 4.99 ± 1.23, and 3.58 ± 1.53 μm, respectively (Fig. 2*D*). A ratio of *V*_max_/*K_m_* defined as catalytic efficiency (Fig. 2*E*) (16, 17) was significantly lower in the R759E mutant (0.16 ± 0.03 μm^−1^ s^−1^) compared with control and suppressor mutant N509K/R759E (0.44 ± 0.11 and 0.37 ± 0.12 μm^−1^ s^−1^, respectively). In contrast, catalytic efficiencies of control and the suppressor mutant were not statistically different. Overall, the suppressor mutant N509K/R759E myosin S1 showed significant enhancement of basal Ca^2+^-ATPase, basal Mg^2+^-ATPase, and actin-stimulated Mg^2+^-ATPase activities compared with converter mutant (R759E) myosin S1; however, most of the ATPase data of the suppressor mutant remain significantly lower than wild-type control values.

##### ATP-induced Dissociation and ADP Affinity of Acto-S1 Are Similar to Wild-type S1 for R759E and R759E/N509K

**FIGURE 3.**
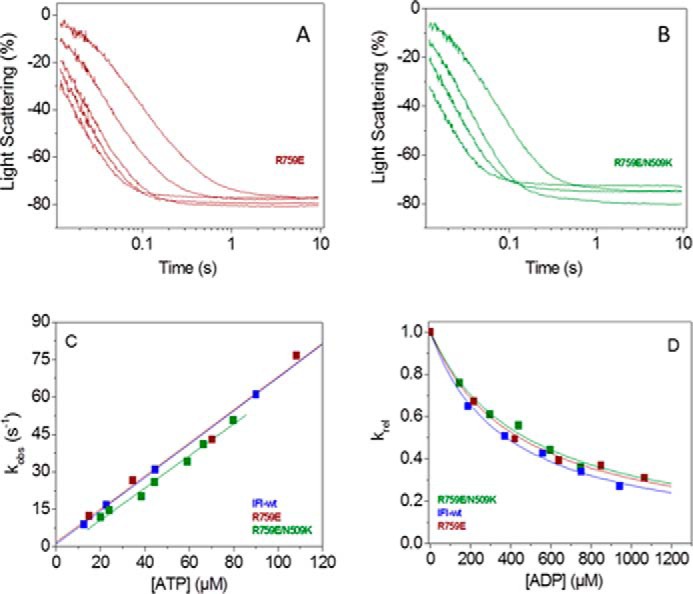
**Summary of transient kinetics measurements of converter mutant and suppressor.** *A* and *B,* example of light scattering traces measured for R759E (*A*) and R759E/N509K (*B*) using flash photolysis and fitted to single exponentials, from which the rate constant *k*_obs_ is determined. *C, k*_obs_ as a function of [ATP] yields the actomyosin dissociation constant *K*_1_*k*_+2_, which is not significantly different for R759E and R759E/N509K compared with wild type. *D,* ATP-induced dissociation of acto-S1 with increasing [ADP] shows similar ADP affinity (*K*_AD_) for R759E and R759E/N509K compared with IFI-WT.

The ATP-induced dissociation of the acto-S1 complex was measured as described previously using flash photolysis to liberate caged ATP (21). Changes in light scattering were recorded and could best be described by a single exponential at each ATP concentration as shown in Fig. 3, *A* and *B,* for R759E and the double mutant R759E/N509K. The slope of a graph of *k*_obs_ *versus* ATP concentration defines the apparent second-order rate constant *K*_1_*k*_+2_ for the dissociation of acto-S1 by ATP. The results for the two mutants together with the IFI wild-type S1 are depicted in Fig. 3*C* and show that the *K*_1_*k*_+2_ values are not significantly different for either of the two mutants compared with wild-type IFI (see Table 1). The ATP-induced dissociation of acto-S1 was also measured in the presence of increasing amounts of ADP to measure the affinity of ADP for actin·myosin, *K*_AD_. Plotting *k*_obs_ *versus* ADP concentration allows *K*_AD_ to be determined, and the results are shown in Fig. 3*D*. The measured values of *K*_AD_ for R759E or R759E/N509K are indistinguishable from the wild-type value (Table 1). Thus both ATP-induced dissociation and ADP-affinity of acto-S1 are not affected for the two mutant myosin proteins.

**TABLE 1:** **Transient kinetic parameters measured for *Drosophila* myosin S1 mutants R759E and R759E/N509K compared with wild-type IFI S1** Values are mean ± S.D. based on a minimum of three preparations.

	Wild-type S1	R759E S1	R759E/N509K S1
**Acto-S1 dissociation*^a^***			
*K*′_1_*k*′_+2_ (μm^−1^ s^−1^)	0.70 ± 0.10	0.72 ± 0.09	0.70 ± 0.05

**ADP affinity of acto-S1*^a^***			
*K*_AD_ (μm)	406 ± 25*^b^*	428 ± 20	421 ± 41

**AD release from S1*^a^***			
*k*_−_*_D_* (s^−1^)	8.0 ± 0.5	7.0 ± 0.4*^c^*	6.0 ± 0.3*^c^*

**ATP binding to S1*^d^***			
*K*_1_*k*_+2_ (μm^−1^ s^−1^)	6.0 ± 0.8	2.7 ± 0.9*^e^*	10 ± 1*^e^*
*k*_+3_ + *k*_−3_ (s^−1^)	308 ± 35	223 ± 28*^e^*	404 ± 111
1/*K*_0.5_ (μm)	53 ± 12	85 ± 30	41 ± 8

*^a^* Measured for this study using flash photolysis.

*^b^ K*_AD_ value is from Ref. 25.

*^c^* Data are significantly different from IFI as determined by the Student's *t* test (*p* < 0.005).

*^d^* Data were measured for this study using stopped flow.

*^e^* Data are significantly different from wild-type S1 as determined by the Student's *t* test (*p* < 0.05).

##### ADP Release from S1-ADP (k_−D_) Is Slower for the Two Converter Mutants R759E and R759E/N509K Compared with Wild-type S1

Using flash photolysis, the rate constant of ADP dissociation from S1 in the absence of actin (*k*_−_*_D_*) can be determined using the fluorescence of a coumarin-labeled ADP (eda-deac ADP). Displacement of eda-deac ADP by ATP binding to S1 results in a fluorescence change from which *k*_−_*_D_* can be estimated. It was shown previously that this coumarin-labeled analogue has very similar kinetic properties to the unlabeled ADP (24). A single laser flash released 15–20 μm ATP from caged ATP (100 μm), and the fluorescence change resulting from eda-deac ADP release is well described by a single exponential function (data not shown). The observed rate constant for ADP release from S1 was slightly reduced (12–25%) for the two converter mutants compared with wild-type S1 (Table 1).

##### Stopped-flow Measurements Show Altered ATP Binding and Hydrolysis Rate Constants by S1 for Both Converter Mutants

**FIGURE 4.**
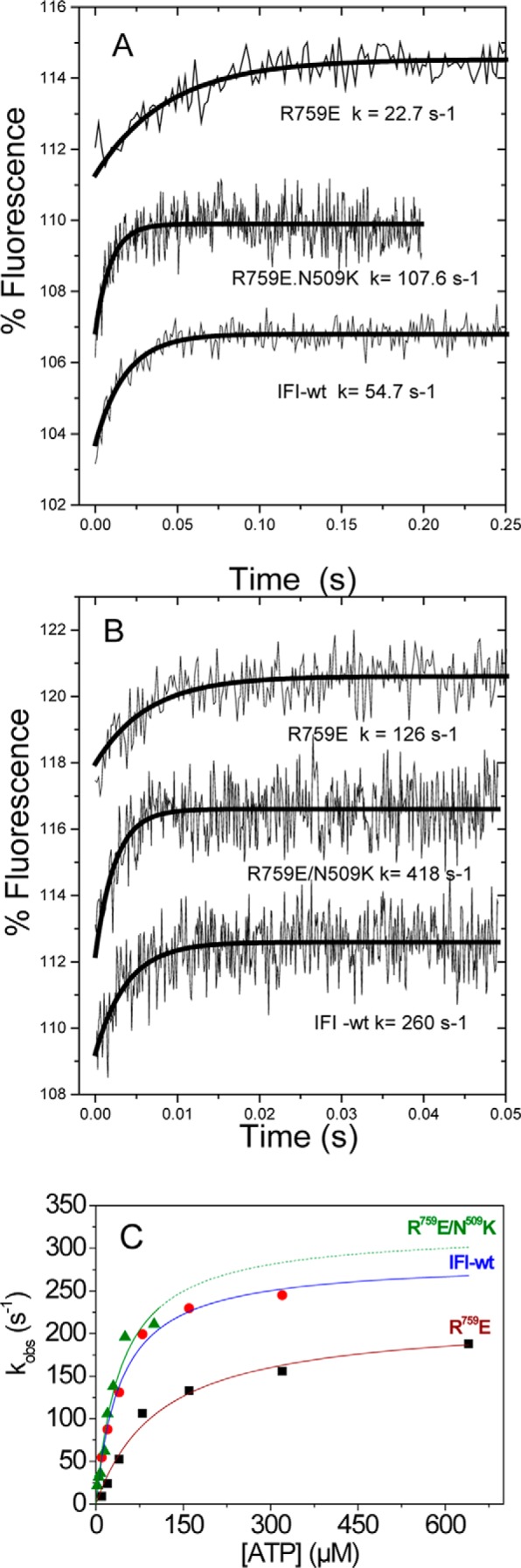
**ATP binding to *Drosophila* S1.** Intrinsic fluorescence transients observed for ATP binding to wild-type myosin S1 and converter mutants R759E and R759E/N509K S1 using stopped flow. 50 nm S1 is rapidly mixed with 10 μm ATP (*A*) or 320 μm ATP (IFI-WT and R759E) or 160 μm ATP (R759E/N509K) (*B*). Note that the amplitudes are small (2–3%) and evaluation of *k*_obs_ values above 200 s^−1^ becomes unreliable. *C,* summary of stopped-flow data: *k*_obs_ as a function of [ATP] yields *k*_max_ = 223 s^−1^ (R759E), 322 s^−1^ (R759E/N509K), and 286 s^−1^ (*IFI-wt*) for a single data set measured for wild-type S1, R759E, and R759E/N509K.

Flash photolysis is usually the method of choice when measuring transient kinetics of *Drosophila* myosin S1, because this method requires much smaller quantities of protein (1 μg) compared with stopped flow. Another reason is that stopped-flow measurements on *Drosophila* myosin were reported to yield relatively poor fluorescence signal changes when using pyrene-labeled actin, whereas light scattering signals are very reproducible (22). However, the laser flash with the caged ATP interferes with intrinsic fluorescence measurements precluding such measurements. The optical performance of stopped-flow systems has improved recently, and we re-examined the binding of ATP to *Drosophila* S1 monitoring intrinsic tryptophan fluorescence. Fig. 4*A* shows the fluorescence change observed on rapidly mixing 10 μm ATP with 0.05 μm wild-type S1 at 20 °C (concentrations are post-mixing). The transient increase in fluorescence is best described by a single exponential with an observed rate constant *k*_obs_ = 54.7 s^−1^ and an amplitude of 3–4%. Example traces of the fluorescence changes at high ATP are shown in Fig. 4*B* and yield an observed rate constant *k*_obs_ = 260 s^−1^ for wild-type myosin S1. The amplitude of the transient was independent of the ATP concentration used. The dependence of *k*_obs_ on ATP concentration for wild-type S1 is shown in Fig. 4*C* with a best fit to a hyperbola superimposed. The fit defines the second-order rate constant of ATP-binding *K*_1_*k*_+2_ = 6.6 ± 0.6 × 10^6^ m^−1^ s^−1^, the maximum observed rate constant at saturating ATP concentrations (average value of *k*_max_ = 286 ± 11 s^−1^, see also Table 1), and the ATP concentration required for the half-maximal *k*_obs_ (*K*_0.5_ = 85 μm).

Repeating this measurement with R759E S1 and R759E/N509K S1 gave similar transients with amplitudes of 3–4% (Fig. 4, *A* and *B*). The ATP dependence of the *k*_obs_ values is shown in Fig. 4*C,* and the average values of all fitted parameters are listed in Table 1. These show that the second-order rate constant for ATP binding to a single mutant R759E (2.7 ± 0.9 × 10^6^ m^−1^ s^−1^) is significantly reduced to about half the value of the IFI-S1 (6.0 ± 0.8 × 10^6^ m^−1^ s^−1^), although for the double mutant this value is significantly increased by about 1.5-fold (10 ± 1 × 10^6^ m^−1^ s^−1^). Similarly the *k*_max_ value is reduced by 25–30% for the single mutant, whereas for the double mutant the value is again ∼1.5 times increased but not significantly different to the wild-type value. In contrast, the value of *K*_0.5_ for the double mutant (41 μm) is very similar to the wild-type value (53 μm), but the value for R759E (85 μm) is nearly double the value of wild-type S1. This is in sharp contrast to the results for actin·S1 where the mutations have little effect on ATP or ADP binding. We must therefore contemplate carefully and exactly what the different fluorescence signals are reporting, and this is considered under the “Discussion.”

##### Interaction between Converter Residue Arg-759 and Relay Residue Asn-509 Is Regulated by SH1 Residue Phe-713

**FIGURE 5.**
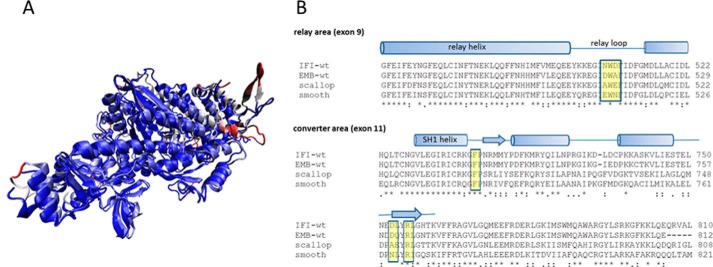
**Myosin head domains of scallop and *Drosophila* are very similar.** *A,* overlay of crystal structures of myosin head domains of scallop (1RS6) and *Drosophila* embryonic myosin (4QBD). The coloring represents the r.m.s.d. values between the two structures with similar structural elements shown in *blue* and deviating structural elements (large r.m.s.d. values) shown in *red*. The backbone of the two structures overlay very well with an average r.m.s.d. < 1.8 Å (shown in *blue*). Flexible loops that diverge somewhat are shown in *red. B,* alignments for *Drosophila* (IFI and EMB), scallop, and chicken smooth myosin. The conserved residues Arg-759 and Phe-713 in the converter area (*bottom*) are highlighted in *yellow*. Note that in regard to the variable residues surrounding the conserved tryptophan (*yellow box* in the relay loop) the Asn-509 residue (*Drosophila* IFI) is replaced by alanine (scallop) and glutamate (chicken smooth).

Homology models were built of wild-type IFI and the two mutants R759E and R759E/N509K. The available scallop myosin-II motor domain crystal structures were initially used as templates, as these represent many different states in the myosin cross-bridge cycle (16). Recently, the first crystal structure of myosin S1 from *Drosophila* (embryonic isoform) has become available (PDB code 4QBD). This structure is very similar to the corresponding scallop crystal structure (PDB code 1SR6), as shown in Fig. 5*A*, with an overall backbone r.m.s.d. of <1.8 Å. The scallop structures have an arginine residue (Arg-754) in the converter domain at the equivalent position as *Drosophila* Arg-759, but in the relay loop area scallop has an alanine instead of asparagine at the equivalent position of *Drosophila* IFI2 Asn-509 (Fig. 5*B*). The homology models show that converter residue Arg-759 interacts with residues located in the relay loop, in the converter, and in the SH1 helix and that, not surprisingly, these interactions depend on the conformational state of the myosin head.

**FIGURE 6.**
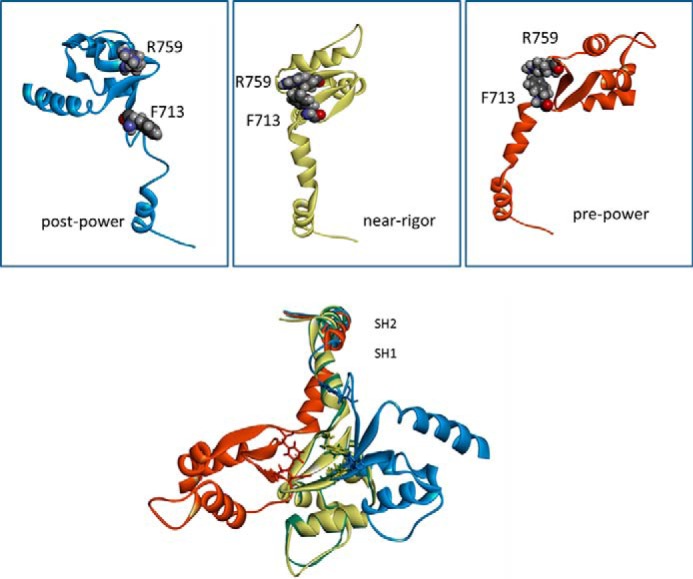
**Interaction between converter residue Arg-759 and SH1 helix residue Phe-713.** *Top panels,* homology models of IFI-WT indicate a strong interaction between Arg-759 and Phe-713 in near-rigor (*yellow*) and pre-power stroke state (*red*), whereas in the post-power stroke state (*blue*) this interaction is not seen, as SH1 has become disordered. *Bottom panel*, overlay of the models for IFI-WT myosin showing the SH1/SH2 and converter area for different myosin conformations. The SH2 area overlays very well but structures start to divert in SH1 area. Residue Arg-759 is indicated for each conformation, together with Phe-713. Homology models of the *Drosophila* IFI myosin isoform were built using the coordinates of scallop myosin PDB 1QVI (pre-power stroke state), PDB 1SR6 (near-rigor state), and PDB 1KK8 (actin-detached post power stroke state) as a templates.

**FIGURE 7.**
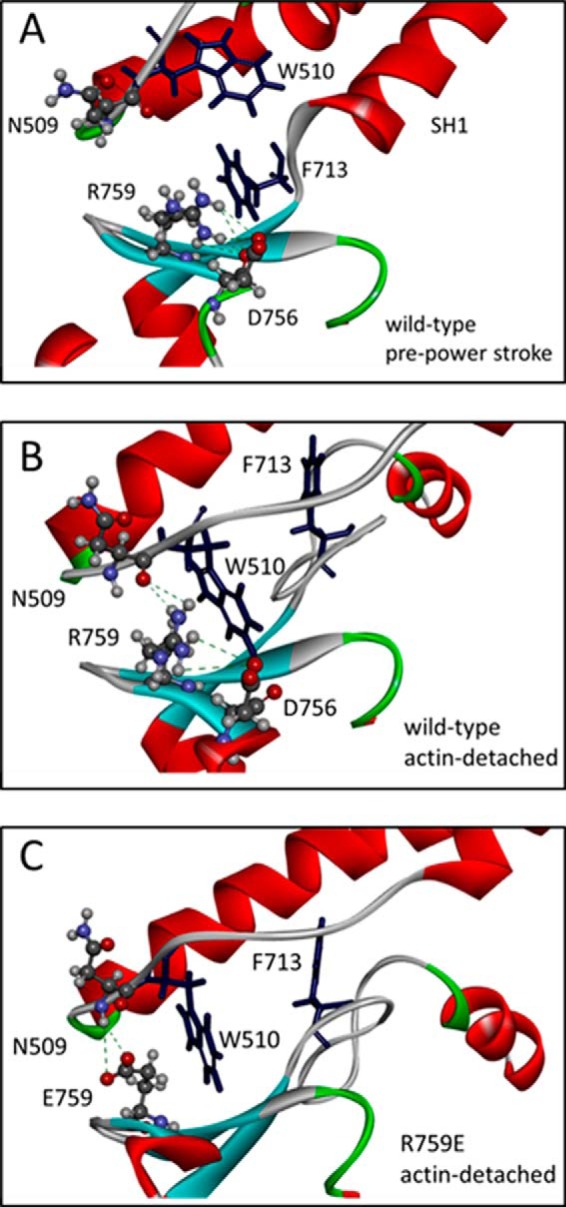
**Details of the relay-converter interaction.** *A,* Arg-759 of wild-type IFI in the pre-power stroke state cannot make contacts with relay loop residues and makes contacts with converter residues (Pro-755/Asp-756) and SH1 residue Phe-713. *B,* in the post-power stroke state (actin-detached), Arg-759 contacts relay residue Asn-509 and Trp-510, in addition to converter residues (Pro-755/Asp-756). Phe-713 has moved away after SH1 helix structure is lost. *C,* R759E mutant residue is able to contact relay loop residue Asn-509 as well in the post-power stroke/actin-detached state. However, additional contacts with converter residues Pro-755/Asp-756 are missing. Homology models were built using scallop myosin PDB 1QVI (pre-power stroke) and PDB 1KK8 (actin-detached, post power stroke state) as a templates.

In the wild-type myosin S1 (IFI-S1) residue Arg-759 forms a salt bridge with Asp-756, another converter residue. This salt bridge is preserved in all homology models of IFI-S1 (Fig. 1*B*) and is also present in the *Drosophila* embryonic myosin crystal structure. Residue Phe-713, located in the SH1 helix, interacts with Arg-759, and this interaction is seen in all models of the myosin states, except the actin-detached state (Fig. 6). The homology models suggest the presence of a π-cation bond between SH1-helix residue Phe-713 and converter residue Arg-759. The presence of a π-cation bond, together with a salt bridge toward Asp-756, holds the Arg-759 side chain oriented toward the converter domain and prevents it from making contact with the relay loop in the pre-power stroke and near-rigor states. In the actin-detached state (1KK8 template), Phe-713 has moved away from Arg-759, as the SH1 helix becomes disordered, and the π-cation bond is lost. Arg-759 now forms strong H-bonds with the backbone C=O of residue Asn-509 in the relay loop while maintaining a salt bridge with converter residue Asp-756 (Fig. 7 and also Fig. 1). The two mutants also show H-bonds between the side chain of residue 759 (Glu-759 for both mutants) and the backbone NH of residue 509 (Asn-509 or Lys-509) in the actin-detached state. However, the mutants are missing the salt bridge seen for wild-type IFI from residue 759 toward Asp-756 (Fig. 7). Thus, the homology models (based on scallop myosin-II templates) suggest that the two mutants have fewer interactions between the converter and relay domain compared with wild-type IFI.

##### Interaction between Converter Residue 759 and Relay Residue 509 Is Disrupted in R759E and Restored in N509K/R759E

**FIGURE 8.**
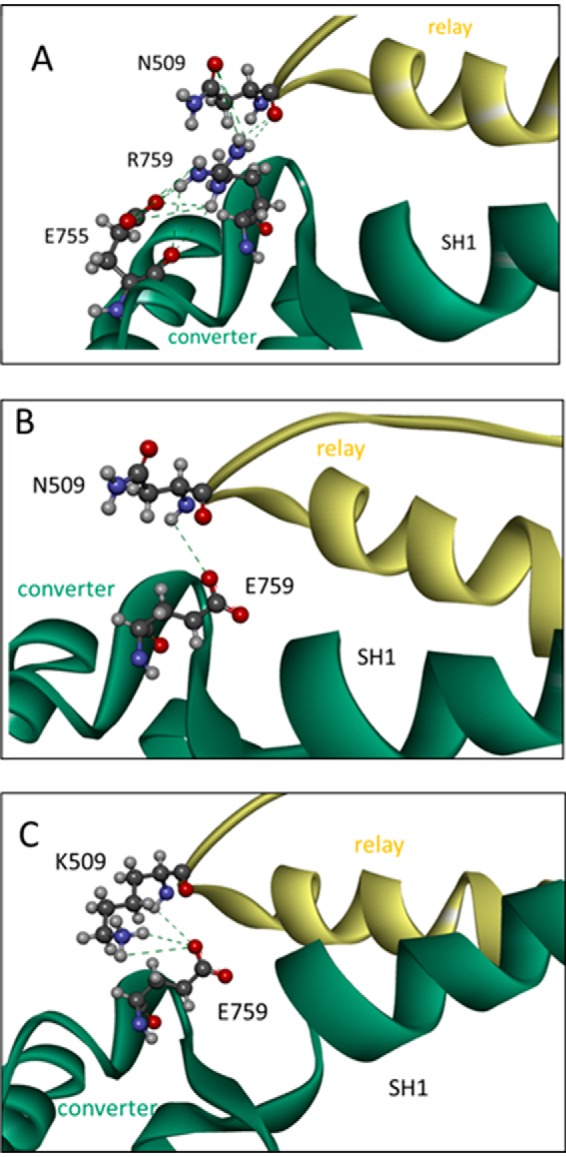
**Side chain interactions between converter residue 759 and relay loop residue 509 for wild-type IFI (*A*), R759E (*B*) and N509K/R759E (*C*).** *A,* close-up of the interface between the relay loop and converter domain with converter residue Arg-759 interacting with the side chain of residue Asn-509, via H-bonds, and converter residue Glu-755 via a salt bridge. *B,* close-up of the interface between the relay loop and converter domain of R759E S1 mutant. The converter residue Glu-759 only forms H-bonds with backbone NH of Asn-509. *C,* close-up of the interface between the relay loop and converter domain of N509K/R759E S1 mutant. The interaction between the side chains of residues 759 and 509 is restored as the side chains are close enough to form hydrogen bonds or a salt bridge. (Homology model was built using the coordinates of chicken smooth muscle myosin in the pre-power stroke state (PDB 3J04) as a template.)

Fewer interactions between the converter and relay loop, as discussed above, can account for the loss of activity seen for R759E but do not provide an explanation for the restored activity in R759E/N509K. Scallop templates, although very useful because of the multiple myosin states available, have an alanine at the equivalent position of Asn-509 of *Drosophila* (Fig. 5*B*) and may therefore not be the best templates to represent the side chain conformation of Asn-509. Chicken smooth muscle myosin has a glutamate (Glu-511) at the equivalent position of Asn-509 (Fig. 5*B*) and could potentially provide a better template to build a homology model that represents the structure of the Asn-509 side chain more accurately. The chicken smooth crystal structures all show a salt bridge between relay residue Glu-511 and Arg-768 (Arg-759 equivalent). Using chicken smooth muscle myosin as a template, we built homology models for wild-type IFI, R759E, and N509K/R759E, and the results are summarized in Fig. 8. For wild-type IFI, the homology model (using the smooth myosin 3J07 structure as template) predicts a direct interaction via hydrogen bonds between the side chains of Arg-759 and Asn-509 within a distance of 2.9–4.5 Å (Fig. 8*A*), which is disrupted when Arg-759 is replaced by Glu-759 (Fig. 8*B*). For N509K/R759E, this interaction is restored, as the homology model predicts the side chains of Glu-759 and Lys-509 are within <3.0 Å and can form a salt bridge (Fig. 8*C*). These observations correlate very well with the loss of activity seen for R759E and the restored activity for N509K/R759E.

## Discussion

The relay-converter interface has been implicated in the communication pathway between the nucleotide-binding site and the lever arm movement (8), and altering this interface can severely disrupt the communication pathway. The interface between the converter and relay helix is believed to be maintained throughout the myosin cross-bridge cycle (9), and its alteration can significantly disrupt the performance of the indirect flight muscle (18) and impair flight ability (19). Additionally, our previous work showed that introducing an R759E mutation in the converter region of full-length myosin reduces ATPase activity and *in vitro* motility and also disrupts flight ability and power generation in *Drosophila* IFM (19). Interestingly, the defects reported for R759E can be suppressed by a second mutation (N509K) in the relay domain. This N509K mutation can restore ATPase activity and *in vitro* motility, and it can also rescue the ability of *Drosophila* carrying R759E to fly (17). Our steady-state kinetics analysis of the isolated S1 fragment verified the effects seen in ATPase activity for full-length myosin. Our transient kinetics analysis sought to identify which step(s) in the cross-bridge cycle are affected by the mutation and the suppressor. Of significance, our data show that the ATP hydrolysis step is strongly affected. This step is known to be coupled to the recovery stroke, which involves the movement of the relay helix and the converter domain (8).

Our transient kinetics data show surprisingly little change in any of the kinetic parameters measured, except those determined in the absence of actin, *i.e.* ATP binding to S1 and its hydrolysis. ATP binding (*K*_1_*k*_+2_) to the single mutant R759E is about half the value found for wild-type S1, although for the double mutant the value is increased by about 1.5-fold of the wild-type value. Similarly, the *k*_max_ value is reduced by 25% for the R759E mutant, whereas for the double mutant the value is higher than found for wild-type S1, although this difference is not statistically significant. The tryptophan changes observed when ATP interacts with myosin S1 can have two components as follows: one from the ATP-binding step (signaled from tryptophan(s) near the nucleotide binding pocket) and another one from the recovery stroke/ATP hydrolysis step signaled by the highly conserved tryptophan at the end of the relay helix (see Fig. 6) that senses the converter movement (35). *Drosophila* myosin has the conserved relay loop Trp-510 next to the mutated Asn-509 and is therefore expected to give a signal change on the recovery stroke/hydrolysis. In contrast, ADP binding gives little change in fluorescence, suggesting little effect on nucleotide binding, and the major contribution of the fluorescence change does originate in the converter domain movement. The standard model for the ATP binding and hydrolysis by myosin is as shown in Reaction 1,

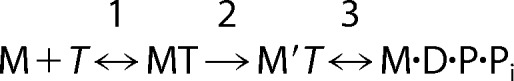
 If the fluorescence change occurs only in step 3 (ATP hydrolysis), then at low [ATP] such that *k*_+3_ + *k*_−3_ ≫ *K*_1_*k*_+2_[ATP], *k*_obs_ has a linear dependence upon ATP concentration, *i.e. k*_obs_ = *K*_1_*k*_+2_[ATP]. At high [ATP] such that *k*_+3_ + *k*_−3_ ≪ *K*_1_*k*_+2_ [ATP], *k*_obs_ is independent of [ATP] and *k*_obs_ = *k*_+3_ + *k*_−3_. Over the full range of ATP concentrations, the dependence of *k*_obs_ on [ATP] approximates to a hyperbola as shown in Fig. 4*C*. A test for such a model is the observed value of the [ATP] required for the half-maximal value of *k*_obs_ (*K*_0.5_ in Table 1). *K*_0.5_ is the ATP concentration at which the *k*_+3_ + *k*_−3_ = *K*_1_*k*_+2_[ATP]. Inspection of the values listed in Table 1 shows this to be true for all three S1 constructs used. Thus we can assign *k*_max_ to *k*_+3_ + *k*_−3_ and the apparent second order rate constant to *K*_1_*k*_+2._ The value of *K*_0.5_ has no specific meaning in the mechanism.

The implications from this analysis are that the mutations are affecting how ATP binds to S1 (*K*_1_*k*_+2_) and also the apparent rate constant of the ATP hydrolysis step *k*_+3_ + *k*_−3_. The change in *K*_1_*k*_+2_ could be due to a change in *K*_1_ or *k*_+2_ or both. Step 1 is the initial binding of ATP into the nucleotide pocket, and step 2 is the induced change in S1 structure associated with switch 1 closure. Any mutation can alter the stability of the apoprotein and potentially disturb the conformation of the binding pocket. Because we do not see any inhibition of ATP binding to acto-S1, it suggests that actin stabilizes the “native-like” structure of the myosin and specifically the nucleotide binding pocket.

Step 3 combines the closure of switch 2, the recovery or re-priming stroke of the myosin head, and the ATP hydrolysis step. The assumption is usually made so that these steps are present for all myosins, and we will make that assumption here. In this model, the closing of the switch 2 loop onto the γ-P_i_ of ATP is required to position the catalytic residues to allow hydrolysis of ATP. At the same time, closure of switch 2 twists the relay helix and results in a change in the position of the converter domain; thereby the lever arm goes through its recovery stroke. Note that Arg-759 on the converter and Asn-509 on the relay helix are part of the structural elements involved in the recovery stroke (7, 36), and mutations here may therefore be expected to influence the hydrolysis step and recovery stroke. The energetics of the recovery stroke and ATP hydrolysis have been well defined for one myosin, the *Dictyostelium* myosin II (8, 37). In *Dictyostelium* myosin II, the equilibrium constants for the recovery stroke and the subsequent hydrolysis step are both quite small (values 0.1–10), and the overall equilibrium constants for the combined step for many muscle myosin IIs are also small, *i.e.* <10. Thus, the system is balanced energetically and hence is susceptible to perturbation by small structural changes anywhere along the pathway from the nucleotide pocket to the lever arm, and many such perturbations have been reported.

Mutations in myosin are instructive in defining domains that are critical for ATPase activity and coupling of the nucleotide pocket to the lever arm. Introduction of point mutations at the base of the myosin lever arm (K84M and R704E) changes the equilibrium constant of the recovery stroke and also alters the ATP hydrolysis rate constant (37). Truncation of the myosin head changed the ATP hydrolysis rate constant in *Dictyostelium* myosin II. Truncations at residues 761, 781, and 864 give myosin fragments with zero (Met-761), one (Met-781), or two (Met-864) light chain binding sites, and the shorter Met-761 fragment displayed the fastest hydrolysis rate constant (160 s^−1^) compared with the longer fragments Met-781 (37 s^−1^) and Met-864 (24 s^−1^) (38). Apart from changes in the hydrolysis rates, the myosins were largely similar to each other. Mutation of two hydrophobic contacts with the converter domain, Ile-499 in the relay loop and Phe-692 in the SH2 region, resulted in uncoupling of the converter rotation and ATP hydrolysis and a complete loss of *in vivo* and *in vitro* motility (39). In other myosin classes, the equilibrium constant for the recovery stroke and ATP hydrolysis may be very different compared with myosin-II. A recently reported crystal structure of myosin-Ib, a very tension-sensitive myosin, revealed a 10-residue stretch of N-terminal amino acids that stabilizes the post-power stroke conformation by making hydrophobic contacts with both the motor and the lever arm helix domain (40, 41). Such contacts have been proposed to play a role in the tension-sensing mechanism of this myosin.

Variations in the converter domain have been found to influence the contractile properties of myosin in muscles. Exchanging the converter domain between the fast IFI and slow EMB isoforms of the *Drosophila* myosin resulted in a shift in contractile properties toward the donor isoform (14). However, this converter swap did not completely exchange the contractile properties between IFI and EMB, suggesting other variable regions in the myosin head contribute as well. Exchange of exon 9 between the IFI and EMB isoforms also significantly affected the mechanical properties of the muscle fibers but again did not result in a complete conversion from EMB to IFI and vice versa (29). Three embryonic myosin isoforms, expressing alternative converter and/or relay domains, were found to have different ATPase activity, *in vitro* motility, and muscle ultrastructure and thus suggested that different forms of relays and converters are used to fine-tune myosin properties (14).

Biochemical studies only assess unloaded transitions in the cross-bridge cycle. Under loaded conditions, the detached states are the same, but the attached part of the cycle may be very different. The evidence from muscle fiber studies is that the converter/relay loop is important for mechanical coupling. Detailed mechanics measurements, using IFM fibers from *Drosophila,* found a significant reduction in muscle power for fibers containing the R759E mutation compared with wild-type fibers (18). The reduced power of R759E IFM fibers was attributed to a decrease in work production due to slower work production, the 2πb component of sinusoidal analysis. No changes were found for the work-absorbing steps (2πc component of sinusoidal analysis), which includes myosin detachment from actin. Our kinetics data show no significant change in steps controlling myosin detachment from actin, ADP-affinity, and ATP-induced dissociation, consistent with no change in the 2πc component from the mechanics study (18). Our experiments cannot assess the work-producing steps of the cycle (2πb); however, the recovery step and hydrolysis step do involve the reversal of the structural changes associated with the power stroke. Notably, the hydrolysis step for R759E was reduced 70% compared with wild-type IFI, while the mechanics study reported a reduction of 68% of the 2πb component for R759E compared with wild-type myosin.

The suppressor R759E/N509K is able to substantially restore *in vitro* motility and myofibril assembly and also rescue flight ability (17), and our results show a significant increase in ATPase activity and ATP binding and hydrolysis compared with R759E, although not to wild-type levels. These observations suggest that an interaction between residue 759 and 509 is necessary for optimal myosin activity. The ability of the double mutation to restore near normal function to the myosin might be interpreted to suggest a salt bridge between the two residues. Our molecular models, using chicken smooth muscle myosin as a template, do predict the presence of a salt bridge between the side chains of residues 759 and 509 in the double mutant. It cannot be excluded that this salt bridge is also formed during other stages of the myosin cross-bridge cycle that are not yet represented in the scallop crystal structures as suggested previously (17).

In conclusion, we find that the interface between relay and converter domain of muscle myosin is critical for optimal myosin performance. Introduction of a mutation (R759E) in the converter domain impairs the steady-state and transient kinetic properties of myosin R759E S1 compared with wild-type S1. Our biochemical approach allows us to identify ATP binding and ATP hydrolysis as the steps that are significantly affected by the R759E mutation and agree with earlier mechanical data for R759E. Introducing a second mutation on the other side of the converter-relay interface in the relay domain (N509K) results in enhanced steady-state and transient kinetics, with a significant increase in ATP binding and hydrolysis for R759E/N509K. Molecular modeling suggests that formation of a salt bridge between these two residues serves as the basis for the observed rescue.

## Author Contributions

M. J. B. performed flash photolysis and stopped-flow and homology modeling work. G. C. M. did the S1 protein purification and ATPase assays. S. I. B. and M. A. G. designed the research. M. J. B., G. C. M., S. I. B., and M. A. G. contributed to data analysis and writing of the manuscript. All authors approved of the final manuscript before submission.

## Footnotes

4 The abbreviations used are:

SW2: switch-2
r.m.s.d.: root mean square deviation
PDB code: Protein Data Bank
eda-deac: 3′-*O*-{*N*-[2-(7-diethylamino-coumarin-3-carboxamido)-ethyl]-carbamoyl ADP
Ap_5_A: *P*^1^,*P*^5^-di(adenosine 5′)-pentaphosphate
IFM: indirect flight muscle
EMB: embryonic
IFI: indirect flight muscle isoform.
